# Separating the Effects of Environment and Space on Tree Species Distribution: From Population to Community

**DOI:** 10.1371/journal.pone.0056171

**Published:** 2013-02-08

**Authors:** Guojun Lin, Diana Stralberg, Guiquan Gong, Zhongliang Huang, Wanhui Ye, Linfang Wu

**Affiliations:** 1 Key Laboratory of Vegetation Restoration and Management of Degraded Ecosystems, South China Botanical Garden, Chinese Academy of Sciences, Guangzhou, People's Republic of China; 2 University of Chinese Academy of Sciences, Beijing, People's Republic of China; 3 Department of Biological Sciences, University of Alberta, Edmonton, Alberta, Canada; CNRS - Université Lyon 1, France

## Abstract

Quantifying the relative contributions of environmental conditions and spatial factors to species distribution can help improve our understanding of the processes that drive diversity patterns. In this study, based on tree inventory, topography and soil data from a 20-ha stem-mapped permanent forest plot in Guangdong Province, China, we evaluated the influence of different ecological processes at different spatial scales using canonical redundancy analysis (RDA) at the community level and multiple linear regression at the species level. At the community level, the proportion of explained variation in species distribution increased with grid-cell sizes, primarily due to a monotonic increase in the explanatory power of environmental variables. At the species level, neither environmental nor spatial factors were important determinants of overstory species' distributions at small cell sizes. However, purely spatial variables explained most of the variation in the distributions of understory species at fine and intermediate cell sizes. Midstory species showed patterns that were intermediate between those of overstory and understory species. At the 20-m cell size, the influence of spatial factors was stronger for more dispersal-limited species, suggesting that much of the spatial structuring in this community can be explained by dispersal limitation. Comparing environmental factors, soil variables had higher explanatory power than did topography for species distribution. However, both topographic and edaphic variables were highly spatial structured. Our results suggested that dispersal limitation has an important influence on fine-intermediate scale (from several to tens of meters) species distribution, while environmental variability facilitates species distribution at intermediate (from ten to tens of meters) and broad (from tens to hundreds of meters) scales.

## Introduction

Understanding the forces driving species distributions is a fundamental goal in ecology, especially with respect to explaining community composition and the maintenance of species diversity [1]–[3]. Both environmental heterogeneity and dispersal limitation have frequently been cited as primary determinants of species distribution [4]–[6]. According to niche theory, species partition resources along environmental gradients and species distributions may therefore be predicted based on environmental factors. There is also increasing support for the importance of non-environmental factors such as dispersal and other population processes in structuring observed species distributions [7], [8]. The expectation in a community driven by dispersal limitation is that the difference in species composition should increase with the distance between communities [9], [10], with species distributions exhibiting spatial aggregation proportionate to dispersal limitation [11].

Recent research has evaluated the relative importance of these two driving forces by comparing the variation in species distribution explained by environmental factors (niche dimensions) to that explained by spatial factors (often assumed to be driven by dispersal limitation). Results have largely shown both types of factors to be important, suggesting that these two processes are both important in determining species' distributions and facilitating species coexistence within a community [9], [12], [13]. However, depending on issues such as data quality and site characteristics, the relative importance of environment and space to species distribution varies considerably across communities and regions. Gilbert and Lechowicz [3] found that species distribution in a North American temperate forest was organized mainly by environmental factors and only secondarily by dispersal events, while Wang et al. [14] found that spatial processes explained much more of the variation in species distribution than environmental processes in a Chinese temperate forest. Results from tropical forest studies also vary substantially [15]. For example, in western Amazonian, Tuomisto et al. [9] found that environmental determinism had a higher explanatory power than dispersal limitation, while Valencia et al. [16] found dispersal limitation to be the main driver of plant species dissimilarity. Clearly, more research is needed to make generalizations about relative contributions of environment and space to species distribution patterns.

Most studies that have quantified the relative importance of environment and space in determining species distributions have only worked at the community level, even though ecological processes act on individuals and populations. Coexisting plant species with different forms and life-history strategies may be differentially constrained by dispersal. For example, overstory species enjoy a height advantage that may help them disperse their propagules more widely, whereas understory species may be more dispersal-limited due to their low height and dispersal resistance [17], [18]. Therefore, understanding the contributing ecological processes at the species level can provide additional insights about community assembly rules.

Understanding the relative effects of environmental and spatial processes on species distribution depends on our ability to decompose the sources of observed variability [12]. The widely used variation partitioning method decomposes total variance of species distribution into the fractions explained by different ecological processes [19] [20]. Although the fraction explained by pure space is usually linked to dispersal processes [3], [21], other spatially structured environmental factors that are not included in the analysis may also contribute [12], [22], leading to an overestimation of the purely spatial fraction [23]. Therefore, further work is needed to identify the relative influence of dispersal limitation [23], [24]. Partitioning the variance of species distributions according to dispersal ability can provide insight into the importance of dispersal processes, especially when environmental factors are strongly structured by space so that they are difficult to decompose completely [9], [25], [26]. In general, species with light propagules or special dispersal organs (wings, hooks, barbs, sticky substances, or fat pulp) can diffuse longer distances and are less dispersal-limited. If dispersal limitation is an important ecological determinant of species distribution, we would expect species with low dispersal ability to have a higher proportion of their variation explained by pure space, compared to species with high dispersal ability.

Environmental conditions are commonly represented by topographic variables alone [16], but edaphic characteristics such as soil moisture and nutrient content also limit plant species distributions. Several landscape-scale studies (>1 km^2^) have demonstrated the importance of soil properties in controlling tree species distributions [27], [28]. However, at local scales (<1 km^2^), the role of edaphic variables in structuring the spatial distribution of trees has not been well studied in natural forests [25], especially in subtropical forests [29]. Furthermore, soil properties are strongly affected by topography [30]. Therefore, further evaluation and partitioning of the effects of soil properties and topography on tree species distribution is an important part of understanding environmental processes.

We aimed to evaluate the relative contributions of niche partitioning and dispersal limitation on species distributions using plant data from a 20 ha stem-mapped subtropical forest plot in Dinghushan, China. Based on plant inventories combined with topographic, edaphic and spatial data, we attempted to disentangle the contributions of environmental and spatial factors at the community and species levels across a range of grain (grid cell) sizes (10-, 20-, 25-, 50-, and 100-m grid cell size). Our specific objectives were 1) to quantify the relative and combined importance of environmental and spatial processes in determining species distribution at the community level and the distributions of species with different life forms; 2) to test the effects of dispersal limitation in determining species distribution, and 3) to further evaluate the role of critical environmental factors (soil and topography) in explaining species distribution. Our goal was to contribute to a larger understanding of the important ecological processes that influence species distribution and promote species coexistence in subtropical forest systems.

## Materials and Methods

### Ethics Statement

No specific permits were required for the described field studies. Our study site (Dinghushan Nature Reserve) is owned by the Chinese government and managed by South China Botanical Garden, Chinese Academy of Sciences. We can do our research works freely in Dinghushan under the Regulations of the People's Republic of China on Nature Reserves. Our field studies did not involve endangered or protected species.

### Study site

**Figure 1:**
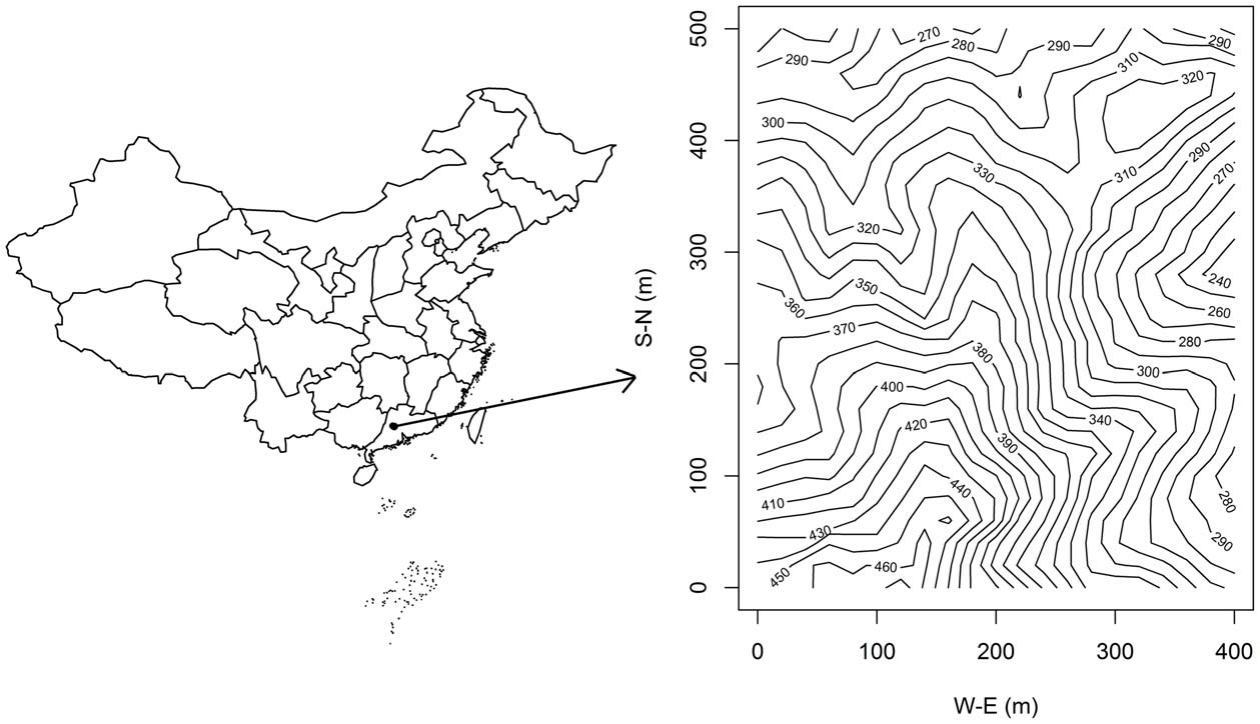
Location and relief map of Dinghushan and the 20 ha stem-mapped permanent forest plot. Values on the relief map represent elevations in meters, which ranges from 230 m to 470 m.

Dinghushan Nature Reserve (23°09′21″–23°11′30″N, 112°30′39″–112°33′41″E, in Guangdong Province, China) (Fig. 1) was established in 1956 as the first nature reserve in China. It contains 1155 ha of subtropical forest. The Dinghushan climate regime is classified as south subtropical monsoon, with a mean annual temperature of 20.9°C, and monthly temperature means varying between 12.6°C and 28°C (January and July, respectively). Mean annual precipitation is 1929 mm, with most precipitation occurring between April and September, while mean annual evaporation is 1115 mm, and relative humidity averages 82%. A permanent 20-ha (400-m×500-m) plot (DHS hereafter) was established in 2005 in the Dinghushan Reserve. The plot is located in rugged terrain and includes three ridges and four valleys, with elevation ranging from 240 to 470 m, as well as complex soil properties. Furthermore, the plot is characterized by an old (>400 yrs), sheltered monsoon evergreen broadleaved forest (Fig. 1). DHS is part of the Chinese Forest Biodiversity Monitoring Network (CForBio), which consists of twelve large (5–25 ha) stem-mapped plots along a latitudinal gradient from tropical to subtropical to temperate forest (http://www.cfbiodiv.org/english/).

### Species data

Following the sampling protocols promoted by the Center for Tropical Forest Science (CTFS) [31], all stems in DHS with diameter at breast height (DBH) no smaller than 1 cm were tagged, georeferenced and identified at the species level. As a result, 71,617 stems belonging to 210 species, 119 genera, and 56 families were recorded (Table S1). Among all species, over 50% (110) can be considered rare in the plot (i.e., with fewer than 20 stems) [32]. *Castanopsis chinensis (Ca.ch), Schima superba (Sc.su), Engelhardtia roxburghiana (En.ro), Syzygium rehderianum (Sy.re), Craibiodendron kwangtungense (Cr.kw), Aidia canthioides (Ai.ca), Cryptocarya chinensis (Cr.ch), Cryptocarya concinna (Cr.co), Aporosa yunnanensis (Ap.yu)* and *Ardisia quinquegona (Ar.qu)* were the ten species with the largest importance values ((relative dominance)/3+(relative frequency)/3+(relative abundance)/3. Relative dominance refers to the basal area of the focal species as a proportion of the total basal area for all species; relative frequency refers to the frequency of the focal species as a proportion of the summed frequency values for all species; relative abundance refers to the abundance of focal species as a proportion of the summed abundance values for all species). Among them, *Ca.ch* was the most dominant overstory species, with 2311 individuals and a maximum DBH of 175 cm and *Cr.ch* was the most dominant species in the midstory, with 2557 individuals and a maximum DBH of 51 cm. Understory species composition in the plot is rich and complex. We counted the number of individuals of each species in each cell for grids of five different height/width dimensions: 10-, 20-, 25-, 50-, and 100-m.

### Environmental characteristics

The topography of the Dinghushan plot was quantified by measuring elevation at the four corners of each cell of a 20-m grid. Elevation values at the 5-m cell size was interpolated by ordinary kriging from 20-m data, while the values for larger cell sizes (i.e., 10-, 25-, 50-, and 100-m) were based on averages of the 5-m cells. For each cell size, we calculated the mean elevation (E), slope (S), convexity (C) and aspect (A) of each grid cell following Harms et al. [26] and Valencia et al. [16]. In order to allow non-linear relationships between topographic factors and response variables, we calculated second-order and third-order polynomials of elevation, slope and convexity. Aspect (degrees from north) was decomposed into east-west and north-south orientation, using sin(aspect) (SA) and cos(aspect) (CA) respectively.

We collected surface-soil samples (0–10 cm) by using a regular grid of points every 30 m. Each alternate grid point was paired with two additional sample points at 2, 5, or 15 m in a random compass direction from the grid to capture fine scale variation in soil properties [25]. In total, 710 samples (four of 714 samples were excluded because they fell on rocks or in creeks) were collected and ten edaphic parameters (moisture (sm), density (sd), pH, organic carbon (tc), available (an) and total nitrogen (tn), available (ap) and total phosphorus (tp), and available (ak) and total potassium (tk)) were measured on each sample. Edaphic variable values at the 5-m cell size was interpolated by ordinary kriging from these measured data, while the values for larger cell sizes (i.e., 10-, 20-, 25-, 50-m and 100-m) were based on averages of the 5-m cell.

### Spatial eigenfunctions

Regardless of their origin, spatial structures cannot be neglected in ecosystems [33], [34]. Principal coordinates of neighbor matrices (PCNM) analysis is a particular case of Moran's eigenvector maps (MEM) analysis [35], an approach that has been proven to better represent spatial patterns than Euclidean distances matrices, geographic coordinates and cubic trend-surface equation [15], [21]. PCNM eigenfunctions are generated by performing a principal coordinate analysis (PCoA) on a truncated Euclidean distance matrix [35]. In our analyses, all distances larger than the distance between the centers of diagonally adjacent cells were replaced by four times that value before PCoA [12]. The axes (eigenvectors) obstained from the PCoA comprise a spectral decomposition of the spatial relationships among the sampling sites, which can be used directly as spatial variables [33]. PCNM eigenfunctions with low ranks represent large scale spatial variation and those with high ranks represent fine-scale spatial variation. More details about how to produce PCNM eigenfunctions can be found in Borcard and Legendre [33] or Dray et al. [35]. We created five sets of PCNM eigenfunctions, each corresponding to a different grid cell size (10-, 20-, 25-, 50-, and 100-m, respectively).

### Variation partitioning analyses

**Figure 2:**
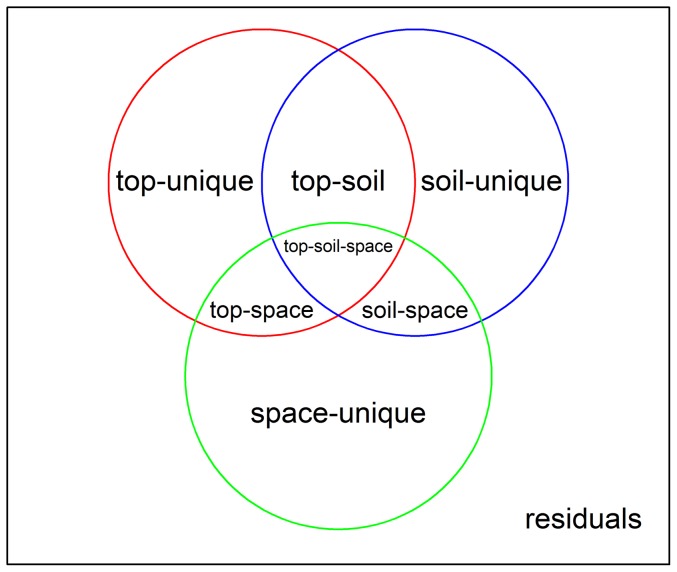
Eight fractions without overlap resulting from variation partitioning using topographic, edaphic and spatial explanatory tables: purely topographic (top-unique), purely edaphic (soil-unique), purely spatial (space-unique), topographically structured edaphic (top-soil), spatially structured edaphic (soil-space), spatially structured topographic (top-space), spatially and topographically structured edaphic (top-soil-space), and unexplained (residuals). Several interpretable quantities can be calculated by adding up fraction values, for example, top-all ( = [purely topographic]+[topographically structured edaphic]+[spatially structured topographic]+[spatially and topographically structured edaphic]) represents the proportion of total variation explained by topography, soil-all ( = [purely edaphic]+[topographically structured edaphic]+[spatially structured edaphic]+[spatially and topographically structured edaphic]) represents the proportion of total variation explained by soil, and space-all ( = [purely spatial]+[spatially structured edaphic]+[spatially structured topographic]+[spatially and topographically structured edaphic]) represents the importance of spatial variables.

At the community level, we conducted variation partitioning based on canonical redundancy analysis (RDA). The overall variation in community composition was divided into fractions attributable to topographic, edaphic and spatial variables, as well as combinations of these explanatory factors. We used the Hellinger-transformed abundance values of species within grid cells as response variables [36]. As partitioning results depend on the scale of analysis, we repeated the same analysis on the five different grid cell sizes (10-, 20-, 25-, 50-m and 100-m). Before conducting variation partitioning, each set of topographic and edaphic variables was standardized and selected at the 95% confidence level using forward selection [37]. Meanwhile, each set of PCNM eigenfunctions was also selected at the 95% confidence level using forward selection [37]. The total variation of species distribution was decomposed into eight fractions (see Fig. 2). Variation partitioning fractions top-unique (purely topographic), soil-unique (purely edaphic) and space-unique (purely spatial) were tested using permutation tests with 999 permutations [19].

Based on RDA analysis, we further analyzed the importance of particular topographic and edaphic factors on species distribution at the community level at the 20-m cell size. We used the Hellinger-transformed abundance values of species at the 20-m cell size as response variables [36]. Standardized topographic and edaphic variables at the 20-m cell size were considered as explanatory variables respectively. For each analysis, we only selected the first three canonical axes to represent the topographic or edaphic variables, because these axes explained more than 80% of the variation. Each canonical axis was tested using a permutation test with 999 permutations [38].

At the species level, we conducted variation partitioning based on multiple linear regressions. For each of the ten species with largest importance value, the overall variation in species distribution was divided into fractions attributable to topographic, edaphic and spatial variables, as well as combinations of these explanatory factors. We used square-root-transformed abundance values of each species within grid cells as response variables [39]. Spatial variables (PCNM eigenfunctions), topographic and edaphic variables were conducted as explanatory variables in the same procedures with that in community level in our analysis.

### Dispersal limitation

We studied dispersal limitation for common species (i.e., those with more than 20 stems in the plot) only, which we divided into three categories according to their dispersal ability (Table S1). Species dispersed by wind and birds were classified as high dispersal (HD), those dispersed by rodents or large mammals (e.g., wild boar) were classified as medium dispersal (MD), and those dispersed by ants or gravity were classified as low dispersal (LD). Species with mixed dispersal modes were classified according to their dominant mode. There were 46, 45, and 9 species in the HD, MD and LD groups, respectively. In order to assess the effect of dispersal limitation, we took the results of variation partitioning obtained for each group at the 20-m cell size and calculated the purely spatial fraction as a proportion of the fraction explained by environment and space combined.

We used the Hellinger-transformed abundance values of each group's species within grid cells as response variables. Other procedures for variation partitioning were the same as for the previous community level analyses in our analysis. We assumed that if dispersal limitation is important in controlling species distributions in Dinghushan plot, the distributions of species with low dispersal ability would be more dependent on spatial variables than the distributions of species with high dispersal ability. That is, the purely spatial fraction would be expected to be higher.

All analyses were conducted using R 2.11.1 [40]. Ordinary kriging was completed using package “gstat” [41]. Forward selection and PCNM eigenfunctions were computed using package “packfor” [42] and “vegan” [38], respectively. Variation partitioning and permutation test were computed using the “vegan” package [38].

## Results

### Variation partitioning by grid-cell size

**Figure 3:**
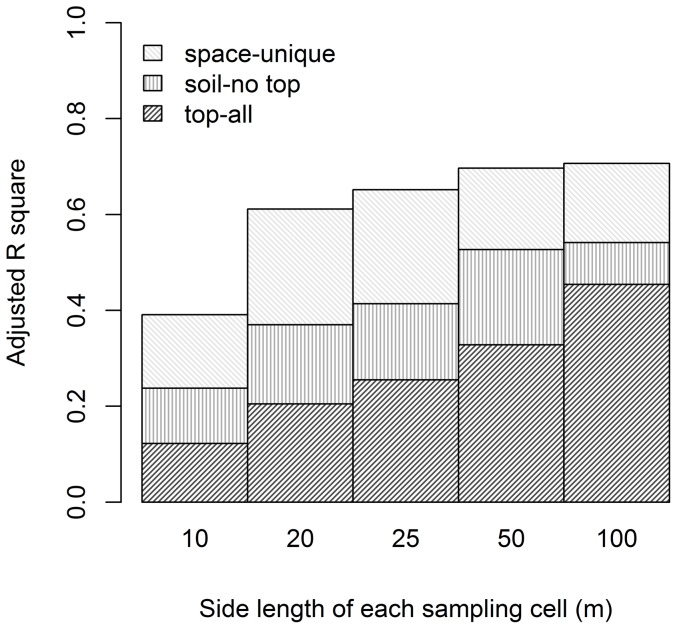
Overall partitioning results for species distribution at the community level. Adjusted R square (Ra^2^) values corresponding to topographic (top-all), edaphic but not topographic (soil-no top), and purely spatial (space-unique) fractions.

The proportion of compositional variation explained increased monotonically with cell size, mainly due to an increase in the environmental fraction. Variation explained by topography and soil increased from 23.7% at the 10-m cell size to 54.1% at the 100-m cell size (Fig. 3). In contrast, the purely spatial (space-unique) fraction was highest at the 20-m cell size (24.1%) (Fig. 3).

**Figure 4:**
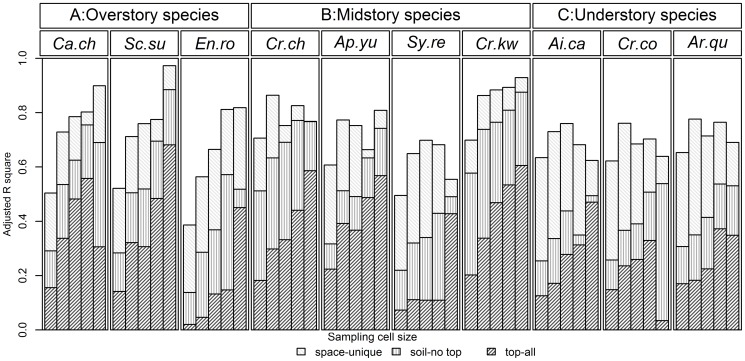
Life forms and variation partitioning results for the ten most abundant species in Dinghushan. The first heading indicates species life forms. The second heading indicates species codes. Ca.ch  = *Castanopsis chinensis*; Sc.su = *Schima superba*; En.ro = *Engelhardtia roxburghiana*; Sy.re = *Syzygium rehderianum*; Cr.kw = *Craibiodendron kwangtungense*; Ai.ca = *Aidia canthioides*; Cr.ch = *Cryptocarya chinensis*; Cr.co = *Cryptocarya concinna*; Ap.yu = *Aporosa yunnanensis*; Ar.qu = *Ardisia quinquegona*. X-axes for each species are the same as in Fig. 3 with five different sampling cell sizes (10-, 20-, 25-, 50-m and 100-m).

Results of variation partitioning on the abundances of each of the ten most important species were generally similar to those obtained for community composition, in that environmental explanatory power generally increased with cell size (Fig. 4). Nevertheless, these ten species were naturally separated into three groups according to their partitioning results. For species of group A (*Ca.ch*, *Sc.su* and *En.ro*), total explanatory power increased gradually with cell size, and the unexplained variation was large in small and intermediate cell sizes. For species of group C (*Ai.ca*, *Cr.co* and *Ar.qu*), total explanatory power was fairly constant across cell size, whereas the purely spatial fraction (space-unique) fraction was large in small and intermediate cell sizes and then decreased gradually with cell size. Finally, species of group B (*Cr.ch*, *Ap.yu*, *Sy.re* and *Cr.kw*) showed patterns that were intermediate between those of groups A and C. Each of these groups represents a different set of life forms. Group A contains only overstory species, whereas group C consists solely of understory species and group B is entirely comprised of midstory species (Fig. 4).

### Environmental fraction

We examined the roles of topography and soil factors in greater depth using the partitioning results obtained at the 20-m cell size. At both the species and community levels, the edaphic (soil-all) fraction was generally larger than the topographic (top-all) fraction (Table 1). Moreover, non-spatial environmental fractions of explained variation (top-unique, soil-unique and top-soil) were relatively small while those with a spatial signature (top-space, soil-space and top-soil-space) provided most of the explanatory power.

**Table 1:** Fractions of variation explained by environmental and spatial variables at the 20-m cell size.

Species	Variation partitioning fractions								
	top-unique	soil-unique	soil-all/top-all	top-unique	soil-unique	top-soil	soil-space	top-space	top-soil-space
CC	0.205	0.289	1.41	0.005	0.003	0.001	0.162	0.076	0.123
*Ca.ch*	0.337	0.466	1.383	0.000	0.014	(0.001)	0.184	0.069	0.267
*Sc.su*	0.321	0.437	1.358	(0.004)	0.013	(0.001)	0.171	0.064	0.252
*En. ro*	0.047	0.280	6.006	(−0.002)	0.006	0.000	0.234	0.008	0.040
*Sy.re*	0.307	0.602	1.962	(0.001)	0.004	(−0.001)	0.322	0.031	0.275
*Cr.kw*	0.392	0.376	0.960	(0.001)	(0.001)	(−0.002)	0.119	0.135	0.258
*Ai.ca*	0.172	0.159	0.926	(−0.002)	(0.001)	0.001	0.164	0.179	−0.006
*Cr.ch*	0.111	0.279	2.498	(0.002)	0.011	(0.002)	0.197	0.040	0.068
*Cr.co*	0.236	0.309	1.309	(−0.001)	(0.003)	0.000	0.128	0.058	0.179
*Ap.yu*	0.338	0.663	1.963	(0.003)	(0.007)	(0.001)	0.394	0.072	0.262
*Ar.qu*	0.183	0.307	1.677	(0.006)	0.000	0.000	0.167	0.038	0.140

Notes: Fractions are the same with that in Fig. 2. top-unique = purely topographic; soil-unique = purely edaphic; top-soil = topographically structured edaphic; soil-space = spatially structured edaphic; top-space = spatially structured topographic; top-soil-space = spatially and topographically structured edaphic; top-all = [purely topographic]+[topographically structured edaphic]+[spatially structured topographic]+[spatially and topographically structured edaphic]; soil-all = [purely edaphic]+[topographically structured edaphic]+[spatially structured edaphic]+[spatially and topographically structured edaphic]. Values in the parentheses for fraction top-unique and soil-unique indicated adjusted R square values (Ra^2^) not significantly different from zero. CC = species distribution at the community level; Ca.ch = *Castanopsis chinensis*; Sc.su = *Schima superba*; En.ro = *Engelhardtia roxburghiana*; Sy.re = *Syzygium rehderianum*; Cr.kw = *Craibiodendron kwangtungense*; Ai.ca = *Aidia canthioides*; Cr.ch = *Cryptocarya chinensis*; Cr.co = *Cryptocarya concinna*; Ap.yu = *Aporosa yunnanensis*; Ar.qu = *Ardisia quinquegona*.

**Figure 5:**
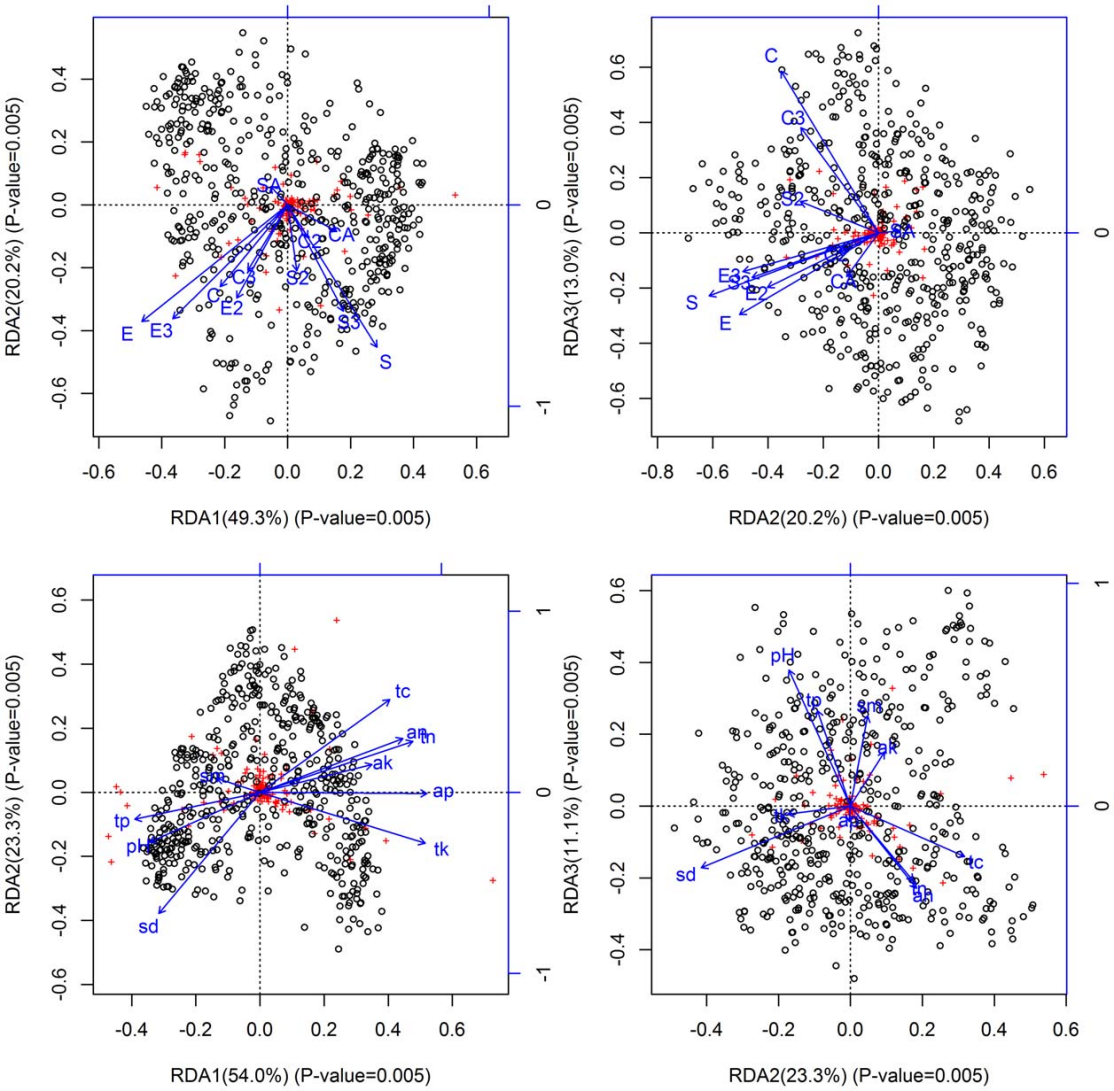
Canonical redundancy analysis (RDA) results at the 20-m cell size for species distribution and environmental factors at the community level. The two graphs in the top panel show relationships between species distribution and topographic factors. E = elevation, S = slope, C = convexity, SA = sin(aspect) and CA = cos(aspect).Associated numbers indicate the power. For example, E2 is the square of elevation. The two graphs in the bottom panel show relationships between species distribution and edaphic factors. sm = soil moisture; sd = soil density; pH = pH; tc = organic carbon; an = available nitrogen; tn = total nitrogen; ap = available phosphorus; tp = total phosphorus; ak = available potassium; tk = total potassium. Values on the coordinate axes represent the proportion of RDA axes. Red crosses in these graphs represent species scores, while black circles represent site scores.

At the community level, RDA conducted using topographic variables at the 20-m cell size resulted in a first canonical axis negatively related to elevation. The second axis mainly represented slope, which was negatively correlated. The third axis mainly represented convexity, which was positively correlated (Fig. 5). Aspect had little influence. Using edaphic variables, the first canonical axis was positively correlated with levels of soil nutrients (e.g., carbon, nitrogen, phosphorus and potassium), except to the total phosphorus. The second axis was mainly related to physical characters of soil (e.g., soil density), whereas the third axis was primarily correlated with pH (Fig. 5).

### Assessment of dispersal limitation

**Figure 6:**
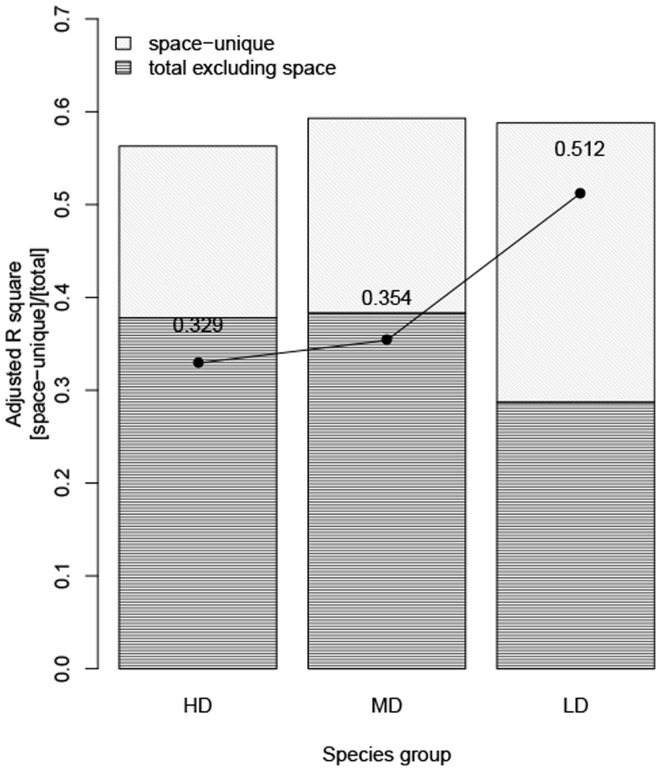
Variation partitioning results at the 20-m cell size for the three species groups in Dinghushan. The X-axis represents different species groups (HD: high dispersal abilities; MD: medium dispersal abilities; LD: low dispersal abilities.). Bars represent adjusted R square values for each partition: purely spatial (space-unique) and [total]-[purely spatial] (total excluding space). Points/lines represent the purely spatial fraction as a proportion of the total fraction explained.

We evaluated the influence of dispersal limitation using the partitioning results obtained at the 20-m cell size (Fig. 6). Total variance explained by the topographic, edaphic and spatial variables was nearly equal across the three species groups, whereas the purely spatial fraction increased gradually as species dispersal abilities decreased. That is the purely spatial fraction as a proportion of the total explained fraction was much higher for species with low dispersal ability than for those with high dispersal ability.

## Discussion

### Scale-dependent ecological processes

At the community level, our variation partitioning analyses revealed scale-dependent controls on species distribution, reflecting the combination of ecological processes that operate at different spatial scales [22], [43]. In the subtropical forest that we studied, environmental variation (i.e., niche partitioning) had strong effects on species distribution at large (from tens to hundreds of meters) and intermediate (from ten to tens of meters) scales, while purely spatial processes, presumed to be driven by dispersal limitation, had non-negligible effects at intermediate scales. At fine scales (from several to ten meters), where unexplained variability was high, direct biotic interactions were presumed to exert an important influence on species distribution.

The importance of environmental variation at large and intermediate scales was consistent with findings from other studies [12], [43], and was not unexpected given the high level of environmental heterogeneity within the site. However, most of the environmental variation was spatially structured (i.e., not possible to separate from spatial factors).

The role of spatial variation, primarily at intermediate scales, was elaborated by our separate evaluation of dispersal limitation, which indicated that species with strong dispersal abilities (i.e., bird- and wind-dispersed species) had lower purely spatial variance components than species with more limited modes of dispersal. Dispersal mode is an important component of dispersal limitation. Thus, there was a large difference between the HD (high dispersal) and LD (low dispersal) groups. However, other aspects such as plant height [17] and pulp-seed attachment [44] may also affect dispersal abilities. In our analysis, we inferred dispersal limitation based on correlational evidence; the difference in the importance of space observed between the high dispersal and medium dispersal groups, while small, was notable. Analysis of plant height and seed rain data may be used to assess the influence of dispersal limitation on species distribution more directly.

Unexplained variation was highest when the grid cell size was small, suggesting the existence of other importance processes for species distribution at fine scales. Li et al. [45] found that self-thinning may influence species' spatial distribution patterns at the scale of 0–10 m. Zhang et al. [46] found evidence of strong interspecific competition at a fine scale (10 m) in temperate forest. We also infer the biotic interactions exert an important influence on fine-scale species distribution patterns in DHS plot. More direct evidence would be helpful, and may be obtained via point pattern analysis or controlled experiments.

### The influence of life form

At the species level, we found that variation partitioning results varied by life form. Overstory and understory species exhibited large differences in the purely spatial fraction at small and intermediate cell sizes. For overstory species, the purely spatial fraction was low, implying that dispersal limitation had little effect on species' distributions at small and intermediate cell sizes. All three overstory species in the DHS plot have high or intermediate dispersal abilities (see Table S1). Therefore, it is reasonable that the distributions of these species are not highly influenced by dispersal limitation. However, the effect of plant height on dispersal limitation cannot be neglected [17]. Distributions of all three understory species were strongly affected by purely spatial factors, implying strong dispersal limitation. Among these three understory species, one (*Ai.ca*) is dispersed by birds. However, its low height and strong dispersal path resistance (i.e., the seed has to cross many obstacles before landing in a suitable area) counteract its dispersal mode advantage. For midstory species, the relative effects of dispersal mode, plant height and other factors could not be identified clearly.

### Relative importance of soil and topography

Compared to topography, we found edaphic factors to explain a greater portion of the variance in species distribution, both at the community and species level. Soil properties directly affect plant growth, and may have stronger explanatory power than topography at local scales. In support of this notion, John et al. [25] found that the distribution patterns of 40% of tree species had strong associations with soil properties at Barro Colorado Island (BCI), and we also found that the percent of variation explained by soil (15%) was higher than that explained by topography (10%) at the 20-m scale within the BCI plot. In another study, Gleason et al. [47] found that most species (59%) showed significant fidelity to specific soil types in a diverse tropical rainforest.

We should also note that edaphic factors could not explain much additional variation in species distribution beyond topographic and spatial information. This does not mean that edaphic and topographic factors are highly correlated (topography only explained 31% of variation in soil properties at the 20-m scale) but that topographic and edaphic variables have similar spatial structuring.

### Edaphic effects

At the community level, soil nutrients explained more of the variation in species distribution than that did other edaphic factors included in our analyses. Pärtel et al. [48], [49] investigated 163 case studies on habitat-productivity-plant-diversity relationships. They found that positive productivity-diversity relationships were common in tropical areas, whereas unimodal (hump-shaped) relationships dominated in the temperate zone. In the subtropical forest plot we studied, as in tropical areas, we found that levels of most nutrients were positively correlated with species distribution. The DHS plot has three salient soil features: (1) nitrogen saturation due to nitrogen deposition and an undisturbed state [50], [51]; (2) limited available phosphorus due to high precipitation, but normal levels of total phosphorus (130–260 mg/kg) [50], [51]; and (3) strong acidification (pH = 3.75±0.20). The low pH value further reduces the availability of potassium and phosphorus, particularly at pH<5 [52]. As a result, potassium and available phosphorus were positively correlated with community composition, while total phosphorus exhibited a negative relationship with community composition. Considering the low pH value in our study area, it is not surprising that pH values were positively correlated with species distribution. A striking finding was that nitrogen was also positively correlated with community composition. We speculate that mature trees are not sensitive to high nitrogen levels, while Lu et al. [51] found that understory diversity decreased with high nitrogen additions (150 kg.ha^−1^.ya^−1^). Soil organic carbon mainly comes from plants, and soil density is correlated with quantity and quality of plant fine roots, so it's not surprising that soil organic carbon and soil density were strongly correlated with species distribution. The DHS forest has ample precipitation. Therefore, compared to soil nutrients, soil moisture did not affect species distribution strongly. According to John et al. [25], trace elements, such as boron, calcium, magnesium, iron and aluminum, may be important for species distribution. We predict that the inclusion of such information would improve the explanatory power of soil characteristics.

### Topographic effects

Elevation, slope and convexity were important topographic factors structuring communities in DHS. Unlike edaphic conditions, the topography of a site influences the growth of plants only indirectly. With increasing elevation, temperature and moisture decrease in the microenvironment and may limit the richness and abundance of plant species. Five general patterns (monotone decrease, single convex peak, single concave, monotone increase and no obvious relationship) have been proposed to describe the relationships between species distribution and elevation [53]; our results support the prevalent notion that species richness and abundance decreases with elevation. Slope limits plant colonization rates, with steep slopes experiencing low rates of diaspore arrival and colonization. Although steeper slopes also include more surface area per plot, potentially accommodating more individual plants, we found more evidence of slope-limited colonization within the DHS plot, in agreement with Wang et al. [30] but not Bin et al. [54]. In DHS, species distribution was also positively correlated with convexity. It is known that convexity affects air humidity, which may explain the positive relationship between species distribution and convexity. A surprising finding was that aspect contributed little to the variance in species distribution in the DHS plot. Aspect mainly corresponds with light requirements; the high abundance of canopy species weakens the selection for light, which may be responsible for this phenomenon.

## Conclusions

In this study, we found evidence that environmental heterogeneity, dispersal and biotic interaction processes worked together to determine species distributions, although their relative importance changed depending on the scale of analysis. Species life form strongly influenced variation partitioning results, with spatial explanatory power highest for understory species and lowest for overstory species. Although soil properties had higher explanatory power than topography, edaphic variables did not contribute much explanatory power beyond topographic and spatial information, due to spatial structuring of these variables. Soil nutrient availability, elevation, slope and convexity were most explanatory of species distributions at the community level.

It is not easy to separate the effects of environment and space on species distribution, even with sophisticated variation partitioning methods. However, our study provided three important new contributions. First, we separated the effects of environment and space on species distribution at the population level, and related it to species life form. We also demonstrated the influence of dispersal limitation on variation partitioning results, rather than merely speculating based on the explanatory power of purely spatial factors. Finally, we explored the role of environmental factors further by comparing the influence of topographic and edaphic factors on species distribution. Our findings improved understanding of species coexistence mechanisms in subtropical forests.

Some limitations of our study should be noted, however. We focused on a single forest plot, and therefore our findings have limited generalizability. Although many studies have attempted to separate the effects of environmental and spatial processes on species distribution patterns, many of them cannot be compared directly due to differences in methods. Thus we advocate the development of integrated research methods in order to produce a unified global dataset from which to study species coexistence mechanisms along elevational, latitudinal, and land-use gradients, to name a few.

Furthermore, as with many variation partitioning studies, our ability to separate environmental and spatial influences was limited. A large portion of the variation in species distribution was explained by spatial and environmental factors together, likely due to highly spatial autocorrelation of environmental factors. We have considered all spatially structured environmental fractions (i.e. top-space, soil-space and top-soil-space fractions in Fig. 2) as niche effects for simplicity, which may lead to an underestimation of spatial processes [55]. However, based on simulated data, Gilbert and Bennett [24] found that ordination using PCNM might under-fit the environmental component and produce inflated R^2^ statistics. They further showed that all of the most commonly employed variation partitioning methods, including the analytical approaches we used, failed to accurately model the spatial and environmental components of variation. Although we conducted an independent supporting analysis (evaluating the influence of dispersal limitation) as suggested by Gilbert and Bennett [24], and we have cautiously interpreted our results, more powerful partitioning techniques should also be developed. In some simple systems, experimental manipulation of ecological processes may help illuminate the relative importance of environmental and dispersal processes on species distribution.

## Supporting Information

Table S1 **Species list for Dinghushan 20 ha permanent forest plot.** The top 100 species are non-rare species and were used in the analysis of dispersal limitation. Importance value (IV) = ((relative dominance)/3+(relative frequency)/3+(relative abundance)/3). Species life forms were classified as overstory, midstory and understory. Species dispersed by wind and birds were classified as high dispersal (HD), those dispersed by mammals (rodents and large mammals such as wild boar and deer) were classified as medium dispersal (MD), and those dispersed by ants or gravity were classified as low dispersal (LD). Dispersal mode was identified by field staff with more than thirty years of experience in the study area.
(DOCX)
