# Supplementary material for: Separating the Effects of Environment and Space on Tree Species Distribution: From Population to Community
Source: PLoS One. 2013 Feb 8;8(2):e56171. doi: 10.1371/journal.pone.0056171 (PMC3568135; doi:10.1371/journal.pone.0056171)
Supplement: Table S1 — Species list for Dinghushan 20 ha permanent forest plot. The top 100 species are non-rare species and were used in the analysis of dispersal limitation. Importance value (IV) = ((relative dominance)/3+(relative frequency)/3+(relative abundance)/3). Species life forms were classified as overstory, midstory and understory. Species dispersed by wind and birds were classified as high dispersal (HD), those dispersed by mammals (rodents and large mammals such as wild boar and deer) were classified as medium dispersal (MD), and those dispersed by ants or gravity were classified as low dispersal (LD). Dispersal mode was identified by field staff with more than thirty years of experience in the study area. (DOCX) [file pone.0056171.s001.docx]

| Species Name | Family | Abundance | IV | Lifeform | Category | Dispersal Mode |
| --- | --- | --- | --- | --- | --- | --- |
| *Castanopsis chinensis* | Fagaceae | 2311 | 12.308 | Overstory | MD | Mammal |
| *Schima superba* | Theaceae | 2296 | 6.600 | Overstory | HD | Wind |
| *Engelhardtia roxburghiana* | Juglandaceae | 737 | 4.771 | Overstory | HD | Wind |
| *Syzygium rehderianum* | Myrtaceae | 5990 | 4.746 | Midstory | HD | Bird |
| *Craibiodendron kwangtungense* | Ericaceae | 3325 | 4.354 | Midstory | LD | Ant, Gravity, Mammal |
| *Aidia canthioides* | Rubiaceae | 5996 | 4.106 | Understory | HD | Bird |
| *Cryptocarya chinensis* | Lauraceae | 2557 | 3.477 | Midstory | MD | Mammal |
| *Cryptocarya concinna* | Lauraceae | 4478 | 3.316 | Understory | MD | Mammal |
| *Aporosa yunnanensis* | Euphorbiaceae | 3747 | 3.005 | Understory | HD | Bird |
| *Ardisia quinquegona* | Myrsinaceae | 3702 | 2.770 | Understory | HD | Bird |
| *Blastus cochinchinensis* | Melastomataceae | 4011 | 2.699 | Understory | LD | Ant, Gravity, Mammal |
| *Acmena acuminatissima* | Myrtaceae | 1484 | 2.553 | Midstory | MD | Mammal, Bird |
| *Ormosia glaberrima* | Papilionaceae | 2702 | 2.225 | Midstory | MD | Mammal |
| *Xanthophyllum hainanense* | Polygalaceae | 1873 | 2.039 | Midstory | MD | Mammal |
| *Lindera metcalfiana* | Lauraceae | 2118 | 1.903 | Midstory | MD | Mammal |
| *Sarcosperma laurinum* | Sarcospermaceae | 1576 | 1.901 | Midstory | LD | Ant, Gravity |
| *Machilus chinensis* | Lauraceae | 532 | 1.739 | Midstory | MD | Mammal |
| *Lindera chunii* | Lauraceae | 1302 | 1.494 | Understory | HD | Bird |
| *Memecylon ligustrifolium* | Melastomataceae | 1263 | 1.365 | Understory | MD | Mammal |
| *Neolitsea umbrosa* | Lauraceae | 1352 | 1.321 | Midstory | HD | Bird |
| *Machilus breviflora* | Lauraceae | 800 | 1.290 | Midstory | HD | Bird |
| *Mischocarpus pentapetalus* | Sapindaceae | 1252 | 1.287 | Understory | MD | Mammal |
| *Canthium dicoccum* | Rubiaceae | 603 | 1.188 | Midstory | HD | Bird |
| *Rapanea neriifolia* | Myrsinaceae | 750 | 1.176 | Understory | HD | Bird |
| *Psychotria asiatica* | Rubiaceae | 908 | 1.160 | Understory | HD | Bird |
| *Rhododendron henryi* | Ericaceae | 810 | 1.077 | Understory | LD | Ant, Gravity |
| *Pinus massoniana* | Pinaceae | 169 | 1.045 | Overstory | MD | Mammal |
| *Garcinia oblongifolia* | Guttiferae | 652 | 1.018 | Understory | MD | Mammal |
| *Ilex triflora* | Aquifoliaceae | 642 | 0.967 | Understory | HD | Bird |
| *Carallia brachiata* | Rhizophoraceae | 724 | 0.945 | Understory | HD | Bird |
| *Artocarpus styracifolius* | Moraceae | 388 | 0.865 | Midstory | MD | Mammal |
| *Eurya macartneyi* | Theaceae | 568 | 0.835 | Understory | HD | Bird |
| *Rhododendron henryi* | Ericaceae | 615 | 0.778 | Understory | LD | Ant, Gravity |
| *Gironniera subaequalis* | Ulmaceae | 286 | 0.757 | Midstory | HD | Bird |
| *Syzygium championii* | Myrtaceae | 404 | 0.750 | Midstory | HD | Bird |
| *Canarium album* | Burseraceae | 332 | 0.680 | Understory | MD | Mammal, Bird |
| *Elaeocarpus sylvestris* | Elaeocarpaceae | 403 | 0.641 | Midstory | HD | Bird |
| *Syzygium levinei* | Myrtaceae | 315 | 0.640 | Midstory | HD | Bird |
| *Diospyros morrisiana* | Ebenaceae | 280 | 0.551 | Understory | MD | Mammal |
| *Archidendron lncidum* | Mimosaceae | 321 | 0.513 | Understory | MD | Mammal |
| *Acronychia pedunculata* | Rutaceae | 231 | 0.497 | Midstory | MD | Mammal |
| *Macaranga sampsoni* | Euphorbiaceae | 675 | 0.489 | Understory | HD | Bird |
| *Ilex chapaensis* | Aquifoliaceae | 136 | 0.483 | Midstory | HD | Bird |
| *Diplospora dubia* | Rubiaceae | 335 | 0.465 | Understory | HD | Bird |
| *Euonymus laxiflorus* | Celastraceae | 269 | 0.462 | Understory | MD | Mammal |
| *Diospyros eriantha* | Ebenaceae | 256 | 0.451 | Understory | MD | Mammal |
| *Schefflera octophylla* | Araliaceae | 198 | 0.440 | Midstory | HD | Bird |
| *Machilus velutina* | Lauraceae | 244 | 0.416 | Understory | MD | Mammal, Bird |
| *Meliosma rigida* | Sabiaceae | 175 | 0.399 | Midstory | MD | Mammal |
| *Symplocos wikstroemiifolia* | Symplocaceae | 116 | 0.374 | Midstory | HD | Bird |
| *Castanopsis fissa* | Fagaceae | 264 | 0.373 | Midstory | MD | Mammal |
| *Nephelium chryseum* | Sapindaceae | 165 | 0.364 | Midstory | MD | Mammal |
| *Litsea verticillata* | Lauraceae | 240 | 0.351 | Understory | MD | Mammal |
| *Pygeum topengii* | Rosaceae | 115 | 0.337 | Midstory | MD | Mammal |
| *Neolitsea membranaceum* | Lauraceae | 223 | 0.298 | Understory | HD | Bird |
| *Photinia prunifolia* | Rosaceae | 114 | 0.291 | Understory | HD | Bird |
| *Ilex memecylifolia* | Aquifoliaceae | 168 | 0.268 | Understory | HD | Bird |
| *Chrysophyllum lanceolatum* | Sapotaceae | 159 | 0.259 | Understory | HD | Bird |
| *Antidesma japonicum* | Euphorbiaceae | 119 | 0.255 | Understory | MD | Mammal |
| *Mallotus paniculatus* | Euphorbiaceae | 146 | 0.210 | Midstory | HD | Bird |
| *Reevesia thyrsoidea* | Sterculiaceae | 177 | 0.207 | Midstory | HD | Wind |
| *Ficus variolosa* | Moraceae | 73 | 0.192 | Understory | HD | Bird |
| *Microdesmis casearifolia* | Euphorbiaceae | 111 | 0.185 | Understory | HD | Bird |
| *Helicia reticulata* | Proteaceae | 73 | 0.176 | Midstory | MD | Mammal |
| *Bridelia fordii* | Euphorbiaceae | 75 | 0.176 | Understory | HD | Bird |
| *Machilus liangkwangensis* | Lauraceae | 72 | 0.160 | Midstory | MD | Mammal |
| *Itea chinensis* | Escalloniaceae | 75 | 0.147 | Understory | MD | Mammal, Bird |
| *Caryota maxima* | Palmaceae | 39 | 0.145 | Midstory | MD | Mammal |
| *Albizia turgida* | Mimosaceae | 65 | 0.142 | Understory | HD | Bird |
| *Melastoma sanguineum* | Melastomataceae | 97 | 0.141 | Understory | MD | Mammal, Bird |
| *Sterculia lanceolata* | Sterculiaceae | 53 | 0.128 | Midstory | MD | Mammal |
| *Evodia lepta* | Rutaceae | 62 | 0.123 | Understory | HD | Bird |
| *Lasianthus chinensis* | Rubiaceae | 48 | 0.116 | Understory | HD | Bird |
| *Casearia villilimba* | Samydaceae | 51 | 0.113 | Midstory | MD | Mammal |
| *Elaeocarpus dubius* | Elaeocarpaceae | 66 | 0.110 | Midstory | MD | Mammal |
| *Ilex ficoidea* | Aquifoliaceae | 48 | 0.110 | Midstory | HD | Bird |
| *Ficus esquiroliana* | Moraceae | 57 | 0.108 | Understory | LD | Ant, Gravity |
| *Gardenia jasminoides* | Rubiaceae | 35 | 0.099 | Understory | HD | Bird |
| *Tarenna mollissima* | Rubiaceae | 39 | 0.098 | Understory | HD | Bird |
| *Elaeocarpus japonica* | Elaeocarpaceae | 50 | 0.097 | Midstory | MD | Mammal |
| *Ormosia fordiana* | Papilionaceae | 63 | 0.094 | Midstory | MD | Mammal |
| *Croton lachnocarpus* | Euphorbiaceae | 48 | 0.093 | Understory | MD | Mammal |
| *Machilus phoenicis* | Lauraceae | 45 | 0.088 | Midstory | HD | Bird |
| *Litsea cubeba* | Lauraceae | 55 | 0.085 | Understory | MD | Mammal |
| *Homalium cochinchinense* | Samydaceae | 36 | 0.082 | Understory | HD | Bird |
| *Mallotus apelta* | Euphorbiaceae | 53 | 0.082 | Understory | HD | Bird |
| *Rhododendron mariae* | Ericaceae | 38 | 0.077 | Understory | LD | Ant, Gravity |
| *Aquilaria sinensis* | Thymelaeaceae | 25 | 0.072 | Midstory | MD | Mammal |
| *Raphiolepis indica* | Rosaceae | 31 | 0.072 | Understory | HD | Bird |
| *Calophyllum membranaceum* | Guttiferae | 28 | 0.069 | Understory | MD | Mammal |
| *Laurocerasus phaeostica* | Rosaceae | 26 | 0.067 | Midstory | MD | Mammal, Bird |
| *Antidesma venosum* | Euphorbiaceae | 27 | 0.065 | Understory | HD | Bird |
| *Syzygium jambos* | Myrtaceae | 28 | 0.062 | Midstory | MD | Mammal |
| *Schoepfia chinensis* | Olacaceae | 24 | 0.057 | Understory | MD | Mammal |
| *Canthium horridium* | Rubiaceae | 24 | 0.056 | Understory | MD | Mammal |
| *Enkianthus quinqueflorus* | Ericaceae | 30 | 0.056 | Understory | LD | Ant, Gravity |
| *Memecylon nigrescens* | Melastomataceae | 27 | 0.056 | Understory | HD | Bird |
| *Pterospermum lanceaefolium* | Sterculiaceae | 22 | 0.045 | Midstory | HD | Wind |
| *Rhododendron simsii* | Ericaceae | 22 | 0.037 | Understory | LD | Ant, Gravity |
| *Cyclobalanopsis jenseniana* | Fagaceae | 28 | 0.024 | Midstory | MD | Mammal |
| *Ilex rotunda* | Aquifoliaceae | 17 | 0.062 | Midstory | - | - |
| *Ormosia semicastrata* | Papilionaceae | 20 | 0.056 | Midstory | - | - |
| *Wikstroemia nutans* | Thymelaeaceae | 19 | 0.051 | Understory | - | - |
| *Zanthoxylum myriacanthum* | Rutaceae | 20 | 0.050 | Midstory | - | - |
| *Glochidion wrightii* | Euphorbiaceae | 19 | 0.048 | Understory | - | - |
| *Antidesma bunius* | Euphorbiaceae | 19 | 0.046 | Midstory | - | - |
| *Pavetta hongkongensis* | Rubiaceae | 17 | 0.041 | Understory | - | - |
| *Sapium discolor* | Euphorbiaceae | 19 | 0.040 | Midstory | - | - |
| *Ficus fistulosa* | Moraceae | 17 | 0.039 | Understory | - | - |
| *Ilex cochinchinensis* | Aquifoliaceae | 12 | 0.036 | Understory | - | - |
| *Vitex quinata* | Verbenaceae | 11 | 0.034 | Midstory | - | - |
| *Syzygium hancei* | Myrtaceae | 10 | 0.031 | Midstory | - | - |
| *Ficus variegata* | Moraceae | 14 | 0.031 | Midstory | - | - |
| *Euonymus nitidus* | Celastraceae | 16 | 0.029 | Understory | - | - |
| *Archidendron clypearia* | Mimosaceae | 10 | 0.026 | Midstory | - | - |
| *Machilus kwangtungensis* | Lauraceae | 7 | 0.024 | Midstory | - | - |
| *Syzygium kwangtungense* | Myrtaceae | 15 | 0.024 | Understory | - | - |
| *Pterospermum heterophyllum* | Sterculiaceae | 14 | 0.023 | Midstory | - | - |
| *Canarium tramdenum* | Burseraceae | 9 | 0.023 | Midstory | - | - |
| *Macaranga bracteata* | Euphorbiaceae | 18 | 0.023 | Understory | - | - |
| *Catunaregam spinosa* | Rubiaceae | 8 | 0.022 | Understory | - | - |
| *Rhododendron tinghuense* | Ericaceae | 10 | 0.021 | Understory | - | - |
| *Rhododendron latoucheae* | Ericaceae | 10 | 0.021 | Understory | - | - |
| *Glycosmis parviflora* | Rutaceae | 8 | 0.021 | Understory | - | - |
| *Eustigma balansea* | Hamamelidaceae | 9 | 0.021 | Understory | - | - |
| *Magnolia paenetalauma* | Magnoliaceae | 12 | 0.020 | Understory | - | - |
| *Ficus nervosa* | Moraceae | 5 | 0.020 | Midstory | - | - |
| *Glochidion eriocarpum* | Euphorbiaceae | 7 | 0.020 | Understory | - | - |
| *Michelia foveolata* | Magnoliaceae | 1 | 0.019 | Midstory | - | - |
| *Alchornea trewioides* | Euphorbiaceae | 14 | 0.018 | Understory | - | - |
| *Cratoxylum cochinchinense* | Hypercaceae | 7 | 0.018 | Understory | - | - |
| *Helicia cochinchinensis* | Proteaceae | 7 | 0.018 | Midstory | - | - |
| *Trema tomentosa* | Ulmaceae | 7 | 0.017 | Understory | - | - |
| *Nauclea officinalis* | Rubiaceae | 5 | 0.017 | Midstory | - | - |
| *Symplocos adenopus* | Symplocaceae | 6 | 0.017 | Midstory | - | - |
| *Indocalamus longiauritus* | Poaceae | 19 | 0.017 | Understory | - | - |
| *Toxicodendron sylvestris* | Anacardiaceae | 6 | 0.015 | Understory | - | - |
| *Casearia glomerata* | Samydaceae | 5 | 0.015 | Midstory | - | - |
| *Wikstroemia indica* | Thymelaeaceae | 6 | 0.014 | Understory | - | - |
| *Ternstroemia gymnanthera* | Theaceae | 7 | 0.014 | Midstory | - | - |
| *Antidesma fordii* | Euphorbiaceae | 13 | 0.014 | Understory | - | - |
| *Litsea rotundifolia* | Lauraceae | 8 | 0.014 | Understory | - | - |
| *Meliosma thorelii* | Sabiaceae | 5 | 0.013 | Midstory | - | - |
| *Symplocos lancifolia* | Symplocaceae | 4 | 0.013 | Understory | - | - |
| *Ilex macrocarpa* | Aquifoliaceae | 5 | 0.013 | Midstory | - | - |
| *Ficus hirta* | Moraceae | 4 | 0.013 | Understory | - | - |
| *Elaeocarpus decipiens* | Elaeocarpaceae | 4 | 0.013 | Midstory | - | - |
| *Ilex pubescens* | Aquifoliaceae | 6 | 0.013 | Understory | - | - |
| *Lindera kwangtungensis* | Lauraceae | 6 | 0.012 | Understory | - | - |
| *Meliosma fordii* | Sabiaceae | 6 | 0.012 | Understory | - | - |
| *Elaeocarpus nitentifolius* | Elaeocarpaceae | 4 | 0.012 | Midstory | - | - |
| *Trema angustifolia* | Ulmaceae | 4 | 0.011 | Understory | - | - |
| *Maesa salicifolia* | Myrsinaceae | 4 | 0.011 | Understory | - | - |
| *Ficus vasculosa* | Moraceae | 3 | 0.010 | Understory | - | - |
| *Symplocos cochinchinensis* | Symplocaceae | 3 | 0.010 | Midstory | - | - |
| *Ormosia pachycarpa* | Papilionaceae | 4 | 0.010 | Midstory | - | - |
| *Adenanthera pavonina* | Mimosaceae | 2 | 0.010 | Midstory | - | - |
| *Aralia spinifolia* | Araliaceae | 5 | 0.010 | Understory | - | - |
| *Podocarpus fleuryi* | Podocarpaceae | 5 | 0.009 | Midstory | - | - |
| *Machilus grijsii* | Lauraceae | 8 | 0.009 | Understory | - | - |
| *Neolitsea aurata* | Lauraceae | 4 | 0.009 | Understory | - | - |
| *Ficus pandurata* | Moraceae | 3 | 0.009 | Understory | - | - |
| *Mallotus hookerianus* | Euphorbiaceae | 3 | 0.008 | Midstory | - | - |
| *Lasianthus curtisii* | Rubiaceae | 3 | 0.008 | Understory | - | - |
| *Neolitsea chuii* | Lauraceae | 3 | 0.008 | Midstory | - | - |
| *Pittosporum glabratum* | Pittosporaceae | 3 | 0.008 | Understory | - | - |
| *Erythrophleum fordii* | Caesalpiniaceae | 2 | 0.007 | Midstory | - | - |
| *Cordia dichotoma* | Boraginaceae | 2 | 0.007 | Understory | - | - |
| *Adinandra millettii* | Theaceae | 3 | 0.007 | Understory | - | - |
| *Microcos paniculata* | Tiliaceae | 2 | 0.007 | Understory | - | - |
| *Ficus superba* | Moraceae | 1 | 0.007 | Midstory | - | - |
| *Securinega virosa* | Euphorbiaceae | 2 | 0.006 | Midstory | - | - |
| *Ormosia semicastrata* | Papilionaceae | 3 | 0.006 | Midstory | - | - |
| *Clerodendrum fortunatum* | Verbenaceae | 2 | 0.006 | Understory | - | - |
| *Aidia pycnantha* | Rubiaceae | 2 | 0.006 | Understory | - | - |
| *Euonymus kwangtungensis* | Celastraceae | 2 | 0.006 | Understory | - | - |
| *Linociera ramiflora* | Oleaceae | 5 | 0.005 | Understory | - | - |
| *Artocarpus tonkinensis* | Moraceae | 1 | 0.004 | Midstory | - | - |
| *Zanthoxylum avicennae* | Rutaceae | 1 | 0.004 | Understory | - | - |
| *Eurya hebeclados* | Theaceae | 3 | 0.004 | Understory | - | - |
| *Daphniphyllum oldhami* | Daphniphyllaceae | 1 | 0.004 | Understory | - | - |
| *Syzygium buxifolium* | Myrtaceae | 2 | 0.003 | Understory | - | - |
| *Sloanea sinensis* | Elaeocarpaceae | 2 | 0.003 | Midstory | - | - |
| *Dimocarpus longan* | Sapindaceae | 2 | 0.003 | Midstory | - | - |
| *Viburnum odoratissimum* | Caprifoliaceae | 2 | 0.003 | Understory | - | - |
| *Eurya nitida* | Theaceae | 1 | 0.003 | Understory | - | - |
| *Garcinia multiflora* | Guttiferae | 1 | 0.003 | Midstory | - | - |
| *Hovenia acerba* | [Rhamnaceae](http://www.efloras.org/florataxon.aspx?flora_id=620&taxon_id=10763) | 1 | 0.003 | Midstory | - | - |
| *Schoepfia jasminodora* | Olacaceae | 1 | 0.003 | Understory | - | - |
| *Styrax suberifolius* | Styracaceae | 1 | 0.003 | Midstory | - | - |
| *Eurya groffii* | Theaceae | 1 | 0.003 | Understory | - | - |
| *Rhodomyrtus tomentosa* | Myrtaceae | 1 | 0.003 | Understory | - | - |
| *Camellia euryoides* | Theaceae | 1 | 0.003 | Understory | - | - |
| *Laportea crenulata* | Urticaceae | 1 | 0.003 | Understory | - | - |
| *Michelia maudiae* | Magnoliaceae | 1 | 0.003 | Midstory | - | - |
| *Cinnamomum camphora* | Lauraceae | 1 | 0.003 | Midstory | - | - |
| *Symplocos chinensis* | Symplocaceae | 1 | 0.003 | Understory | - | - |
| *Ehretia longiflora* | Boraginaceae | 1 | 0.003 | Understory | - | - |
| *Clerodendrum cyrtophyllum* | Verbenaceae | 1 | 0.003 | Understory | - | - |
| *Clerodendrum japonicum* | Verbenaceae | 1 | 0.003 | Understory | - | - |
| *Eurya chinensis* | Theaceae | 1 | 0.003 | Understory | - | - |
| *Litchi chinensis* | Sapindaceae | 1 | 0.003 | Midstory | - | - |
| *Pentaphylax euryoides* | Pentaphylaceae | 1 | 0.003 | Midstory | - | - |
| *Mallotus philippensis* | Euphorbiaceae | 1 | 0.003 | Understory | - | - |
| *Eriobotrya fragrans* | Rosaceae | 1 | 0.003 | Midstory | - | - |
| *Callicarpa longifolia* | Verbenaceae | 1 | 0.003 | Understory | - | - |
| *Glochidion puberum* | Euphorbiaceae | 1 | 0.003 | Understory | - | - |
| *Viburnum sempervirens* | Caprifoliaceae | 1 | 0.003 | Understory | - | - |
| *Lindera communis* | Lauraceae | 1 | 0.003 | Midstory | - | - |
| *Ixora chinensis* | Rubiaceae | 1 | 0.003 | Understory | - | - |
